# Antioxidant and Anti-Inflammatory Effects of *Bischofia javanica* (Blume) Leaf Methanol Extracts through the Regulation of Nrf2 and TAK1

**DOI:** 10.3390/antiox10081295

**Published:** 2021-08-16

**Authors:** Sewoong Lee, Jain Ha, Jiyoung Park, Eunjeong Kang, Sung-Hyun Jeon, Sang Beom Han, Sri Ningsih, Jin Hyub Paik, Sayeon Cho

**Affiliations:** 1 Laboratory of Molecular and Pharmacological Cell Biology, College of Pharmacy, Chung-Ang University, Seoul 06974, Korea; dltpdnd2000@naver.com (S.L.); joehalee@gmail.com (J.H.); cynical-0528@hanmail.net (J.P.); ejaykang@gmail.com (E.K.); 2 Biomedical Mass Spectrometry Lab, College of Pharmacy, Chung-Ang University, Seoul 06974, Korea; rkwhr9068@naver.com (S.-H.J.); hansb@cau.ac.kr (S.B.H.); 3 Center for Pharmaceutical and Medical Technology, Deputy for Agroindustrial Technology and Biotechnology, The Agency for the Assessment and Application of Technology (BPPT), Jl. Raya Puspiptek, Kota Tangerang Selatan 15310, Banten, Indonesia; sri.ningsih@bppt.go.id; 4 International Biological Material Research Center, Korea Research Institute of Bioscience and Biotechnology, Daejeon 34141, Korea; jpaik@kribb.re.kr

**Keywords:** *Bischofia javanica* Blume, RAW 264.7 macrophages, antioxidant, anti-inflammation, nitric oxide, nuclear factor-κB, nuclear factor erythroid 2-related factor 2

## Abstract

*Bischofia javanica* (Blume) has been traditionally used to treat inflammatory diseases such as tonsillitis and ulcers throughout Asia, including China, Indonesia, and the Philippines: however, the molecular mechanisms by which *B. javanica* exerts its antioxidant and anti-inflammatory properties remain largely unknown. In this study, we analyzed the antioxidant and anti-inflammatory mechanisms of methanol extracts of *B. javanica* leaves (MBJ) in vitro and in vivo. MBJ decreased nitric oxide (NO) production and the expression of pro-inflammatory cytokines, including interleukin (IL)-1β, IL-6, and tumor necrosis factor-α, in lipopolysaccharide (LPS)-treated RAW 264.7 cells. The observed suppression of inflammatory responses by MBJ was correlated with an inhibition of the nuclear factor-κB (NF-κB) and the mitogen-activated protein kinase (MAPK) pathways. Additionally, MBJ induced nuclear translocation of the nuclear factor erythroid 2-related factor 2 (Nrf2), a transcription factor that upregulates the expression of anti-inflammatory and antioxidant genes. Furthermore, MBJ exhibited antioxidant and anti-inflammatory effects in an acute hepatitis mouse model. In conclusion, our results confirm the medicinal properties of *B. javanica*, and therefore MBJ could be applied to improve inflammatory and redox imbalances in different types of pathologies.

## 1. Introduction

The inflammatory response defends hosts from harmful external factors such as bacteria and fungi [1], and this process is largely mediated by innate immune cells [2]. For example, macrophages become activated when they recognize external antigens such as lipopolysaccharides (LPS; i.e., the constituents of Gram-negative bacterial cell walls) [3]. In turn, the activated macrophages trigger inflammation by producing pro-inflammatory mediators such as tumor necrosis factor (TNF)-α, interleukin (IL)-1β, IL-6, and nitric oxide (NO) [4,5]. In addition, oxidative stress occasionally leads to chronic inflammatory diseases [6]. Oxidative stress and inflammation are interdependent mechanisms, as reactive oxygen species (ROS) trigger cytokine release, and the excessive production of cytokines induces oxidative stress [7]. The nuclear factor erythroid 2-related factor 2 (Nrf2) plays a pivotal role in protecting cells against oxidative stress. Nrf2 functions as a transcription factor by translocation to the nucleus in response to oxidative stress to induce the expression of antioxidant genes such as heme oxygenase-1 (*HMOX1*), glutamate—cysteine ligase catalytic subunit (*GCLC*), and NAD(P)H dehydrogenase [quinone] 1 (*NQO1*) [8]. In turn, the uncontrolled production of inflammatory mediators causes inflammatory diseases, such as asthma, rheumatoid arthritis, tuberculosis, and ulcers [9,10]. Therefore, proper regulation of activated macrophages is a necessary step toward the development of therapeutic strategies for the treatment of inflammatory diseases.

When Toll-like receptor 4 (TLR4) is stimulated by LPS, IL-1 receptor-associated kinase 1 (IRAK1) of the receptor complex undergoes phosphorylation-mediated degradation, which induces the release and phosphorylation of transforming growth factor-β-activated kinase 1 (TAK1) [11]. TAK1 is activated via dual phosphorylation at the Thr184/187 residues, then triggers the activation of the nuclear factor-κB (NF-κB) and mitogen-activated protein kinase (MAPK) pathways [12,13]. While the MAPK pathway induces activation of downstream transcription factors such as activator protein 1 (AP-1), NF-κB directly functions as a transcription factor as it translocates to the nucleus [14]. In the nucleus, AP-1 and NF-κB induce the expression of inducible NO synthase (*iNOS*) and cyclooxygenase-2 (*COX-2*) [15]. The expressed iNOS and COX-2 catalyze NO synthesis and the production of prostaglandins (PGs), respectively, which are upregulated in immune cells showing inflammatory responses [16,17]. Furthermore, inflammatory cytokines are related to several inflammatory diseases. In particular, the dysregulation of IL-1β can lead to chronic autoimmune diseases of the central nervous system, such as rheumatoid arthritis [18,19]. *IL-6* synthesis is the major driver of the initial stage of inflammation and inflammatory diseases, whereas *TNF-α* promotes inflammatory responses in the human body and its expression is associated with rheumatoid arthritis [20,21]. Therefore, using natural products to suppress the inflammation-promoting signaling pathways is a promising strategy for treating inflammatory diseases.

*Bischofia javanica* (Blume), which belongs to the family Phyllanthaceae, has been widely applied as a traditional medicine throughout China, Indonesia, and the Philippines [22,23]. The young leaves of *B*. *javanica* are used to treat sores, tonsillitis, and throat pain [24,25]. The juices of the leaves are used to promote the healing of skin severe wounds, and the bark of *B. javanica* is used for the treatment of tuberculosis, stomach ulcers, and mouth ulcers [26]. Given the effectiveness of *B*. *javanica* in traditional medicine, the constituents and effects of *B*. *javanica* have been recently analyzed in an effort to identify its active components and mechanisms of action. The major phytoconstituents of *B*. *javanica* are tannins, β-amyrin, betulinic acid, luteolin, quercetin, β-sitosterol, stigmasterol, and ursolic acid [27]. Triterpene ursolic acid and steroid β-sitosterol found in *B. javanica* were shown to suppress COX-1 activity [28]. Additionally, an extract of *B. javanica* leaves exhibited anti-inflammatory effects against acute carrageenan-induced paw edema in rats [29]. Although *B. javanica* extracts have been found to possess anti-inflammatory effects and many of their chemical constituents have been identified, the molecular mechanisms by which *B*. *javanica* exerts its antioxidant properties in macrophages remain largely uncharacterized. Therefore, the antioxidant and anti-inflammatory mechanisms of a methanol *B. javanica* leaf extract (MBJ) were investigated by evaluating its effects on the intracellular signaling pathways and inflammatory mediators of LPS-stimulated RAW 264.7 cells.

## 2. Materials and Methods

### 2.1. Preparation of MBJ

Fresh *B. javanica* leaves were collected from the Alas Purwo National Park, East Java, Indonesia. Plant samples were collected and identified by staff at the Center for Pharmaceutical and Medical Technology (PTFM; Tangerang, Indonesia), and verified at the Herbarium Bogoriense (LIPI; Bogor, Indonesia). According to the International Union for Conservation of Nature, *B. javanica* is among the species of least concern as of 20 September 2018 [30]. To prepare methanol extract of *B. javanica* (MBJ), a total of 554.42 g of powder-dried leaves of *B. javanica* was extracted with 1.5 L of methanol with agitation for 1 h and left for a night at room temperature (RT). Then, the methanol extract was filtered and extraction was repeated twice. The collected filtrate was concentrated using a rotary evaporator (Rotavapor 4000; Heidolph, Schwabach, Germany) until semisolid mass was obtained. The Bischofia javanica Blume leaves extract was kept in a sealed dark-glass container for further use. Voucher specimens recorded as KRIB 0039625 and PMT 1349, were deposited in the herbarium of the Korea Research Institute of Bioscience and Biotechnology (KRIBB; Daejeon, Korea) as well as in the Center for Pharmaceutical and Medical Technology (PTFM) and the Herbarium Bogoriense. In our experiments, a semi-solid mass of MBJ was dissolved in dimethyl sulfoxide (DMSO; Sigma-Aldrich, St. Louis, MO, USA) and added to the culture media to the final concentration indicated in each experiment. To avoid cell damage, the final concentration of DMSO never exceeded 1% in all experiments.

### 2.2. Antibodies

Rabbit polyclonal anti-iNOS (cat no. 2982), goat polyclonal anti-COX-2 (cat no. sc-4842), mouse monoclonal anti-GAPDH (cat no. sc-47724), mouse monoclonal anti-c-Jun N terminal kinase (JNK; cat no. sc-7345), rabbit polyclonal anti-phospho (p)-inhibitor of κBα (IκBα) (Ser32/36; cat no. sc-371), mouse polyclonal anti-p38 (cat no. sc-7972), rabbit polyclonal anti-IκBα (cat no. sc-7607), mouse monoclonal anti-IRAK1 (cat no. sc-5288), and mouse monoclonal anti-α-tubulin (cat no. sc-5286) antibodies were purchased from Santa Cruz Biotechnology, Inc. (Santa Cruz, CA, USA). Rabbit polyclonal anti-p-p38 (Thr180/Tyr182; cat no. 9211), rabbit polyclonal anti-extracellular signal-regulated kinase (ERK; cat no. 9102), rabbit polyclonal anti-p-JNK (Thr183/Tyr185; cat no. 9252), rabbit monoclonal anti-p-ERK (Thr202/Tyr204; cat no. 9106), rabbit polyclonal TAK1 (cat no. 4505), rabbit monoclonal anti-p-TAK1 (Thr184/187; cat no. 4508), rabbit monoclonal anti-Lamin B1 (cat no. 12586), and rabbit monoclonal anti-Nrf2 (cat no. 12721) antibodies were purchased from Cell Signaling Technology Inc. (Danvers, MA, USA). Primary antibodies were diluted in 5% nonfat-dried skim milk or 5% bovine serum albumin in 1× TBST solution at a 1:1000 ratio. Polyclonal anti-rabbit IgG Fc-HRP (cat no. LF-SA8002) and polyclonal anti-mouse IgG Fc-HRP (cat no. LF-SA8001) were from AbFrontier (Seoul, Korea). Secondary antibodies were diluted in 5% nonfat-dried skim milk in TBST at a 1:5000 ratio. The Ready-SET-Go! ELISA kits for the detection of IL-6 (cat no. 88-7064), IL-1β (cat no. 88-8013), and TNF-α (cat no. 88-7324) were obtained from eBioscience (San Diego, CA, USA).

### 2.3. High-Performance Liquid Chromatography (HPLC) Analysis

Standard stock solutions of MBJ, quercetin, and luteolin, were dissolved in a 5 mg/mL mixed solution composed of 0.1% aqueous formic acid in water and 1% formic acid in acetonitrile (70/30, *v/v*). All standard solutions were filtered through a 0.45 µm syringe filter and analyses were performed using an Agilent 1260 HPLC system with a diode array detector. The detection wavelength was set at 360 nm.

The analyses were conducted using a Phenomenex Kinetex C18 reversed-phase column (4.6 mm × 250 mm, 5 µm particle size) at 25 °C. The mobile phase consisted of 0.1% formic acid in water (A) and 0.1% formic acid in acetonitrile (B) at a flow rate of 1.0 mL/min. Gradient elution was performed as follows: 30% (B) for 0–5 min, 30–50% (B) for 5–40 min, 50% (B) for 40–41 min, 50–95% (B) for 41–42 min, and finally the elution was held at 95% (B) for 2 min. The injection volume was 10 µL.

### 2.4. Cell Culture and Reagents

RAW 264.7 macrophages and HEK 293 cells were purchased from the American Type Culture Collection (Manassas, VA, USA). Cells were maintained in Dulbecco’s Modified Eagle’s Medium (DMEM; Invitrogen, Carlsbad, CA, USA) with 10% fetal bovine serum (FBS; Invitrogen) and 1% penicillin/streptomycin (Life Technologies, Carlsbad, CA, USA) at 37 ℃ in the presence of 5% CO_2_. All of the assays that involved live cells were conducted at 37 ℃ incubation temperature unless explicitly described otherwise. LPS was purchased from Sigma-Aldrich. *IRAK1/4* inhibitor (cat no. 5665) was purchased from Toris Bioscience (Avonmouth, UK).

### 2.5. Cell Viability Assay

RAW 264.7 macrophages were seeded onto 96-well plates at 4.5 × 10^4^ cells/well density and treated with MBJ (20, 50, 100, and 150 μg/mL) for 2 h, then stimulated with LPS (1 μg/mL) for 24 h. Cytotoxic effects were measured using an EZ-Cytox cell viability assay kit (Daeil Lab, Seoul, Korea). The EZ-Cytox solution was added to the cell culture (1/10 culture medium) and then incubated for 1 h. Cell viability was calculated based on the absorbance of viable cells at 450 nm and the reference absorbance at 650 nm (A_450_–A_650_) using a Synergy H1 Microplate Reader (BioTek Instruments, Inc., Winooski, VT, USA).

### 2.6. Nitrite Assay

RAW 264.7 macrophages were seeded onto 96-well plates (4.5 × 10^4^ cells/well) and incubated overnight. Cells were incubated with MBJ (20, 50, 100, and 150 μg/mL) for 2 h and then with LPS (1 μg/mL) for 24 h. Afterward, 100 μL of the culture media was transferred to a new 96-well plate and then mixed with 100 μL of Griess reagent (0.1% *N*-(1-naphthyl) ethylenediamine, 2.5% phosphoric acid (H_3_PO_4_), and 1% sulfanilamide in distilled water). Sodium nitrite was used to generate a standard curve. The absorbance was measured at 540 nm using Synergy H1 Microplate Reader (BioTek Instruments, Inc., Winooski, VT, USA).

### 2.7. Reverse Transcription-Polymerase Chain Reactions (RT-PCR)

RAW 264.7 macrophages were seeded onto 12-well plates (2 × 10^5^ cells/well) and incubated overnight. The cells were then pre-treated with MBJ (20, 50, 100, and 150 μg/mL) for 2 h and stimulated with LPS (1 μg/mL) for 3 h. Total RNA was extracted using the Accuzol total RNA extraction reagent (Bioneer Corporation, Daejeon, Korea). The total RNA (1 μg) was then reverse transcribed into complementary DNA (cDNA) using a TOPscript cDNA synthesis kit (Enzynomics, Daejeon, Korea). The amplification conditions were the following: 95 °C (5 min) initial denaturation followed by 25 cycles of 95 °C for 5 s and annealing/extension at 55 °C (30 s). The gene expression levels were normalized relative to those of the reference gene glyceraldehyde 3-phosphate dehydrogenase (*GAPDH*).

### 2.8. Reverse Transcription-Quantitative Polymerase Chain Reactions (RT-qPCR)

RAW 264.7 macrophages were seeded in 12-well plates (2 × 10^5^ cells/well) and incubated overnight. Cells were pre-treated with MBJ (20, 50, 100, and 150 μg/mL) for 2 h and stimulated with LPS (1 μg/mL) for 3 h. Total RNA extraction and cDNA synthesis were carried out as described above. iTaq Universal SYBR-Green Supermix was used to amplify the cDNA according to the manufacturer’s instructions. The amplification conditions were the following: 95 °C (5 min) initial denaturation followed by annealing/extension at 60 °C (30 s) using a CFX Connect real-time thermal cycler (Bio-Rad Laboratories, Inc. Hercules, CA, USA). The gene expression levels were normalized to those of *GAPDH* (i.e., the reference gene) and were reported as percentages of the control group (assuming that the control expression was 100%) following the 2^−ΔΔCq^ method. The sequences of the PCR primers used herein are the same as those listed in a previous study [31].

### 2.9. Enzyme-Linked Immunosorbent Assay (ELISA)

RAW 264.7 macrophages were seeded onto 96-well plates (4.5 × 10^4^ cells/well) and incubated overnight. The cells were treated with MBJ (20, 50, 100, and 150 μg/mL) for 2 h and then with LPS (1 μg/mL) for 24 h. Culture supernatants were then collected and the concentrations of IL-6, IL-1β, and TNF-α were measured using sandwich ELISA with monoclonal antibodies specific to each mediator according to the manufacturer’s instructions. In brief, 96-well ELISA plates were pre-coated with the capture antibody at 4 °C overnight. The plate was then washed four times with 1× phosphate-buffered saline/0.05% Tween 20 (PBST) and blocked with 1× assay diluent at RT for 1 h. The sample (100 μL) was then added to each well and incubated at RT for 2 h. Following the incubation, a biotinylated detection antibody solution was added at RT for 1 h. The plate was then treated with horseradish peroxidase (HRP)-streptavidin solution at RT for 30 min. Afterward, 100 μL of 3,3′,5,5′-tetramethylbenzidine (TMB) was added at RT for 10 min under dark conditions. Next, 50 μL of 1M H_3_PO_4_ was added to each well to stop the reaction. The absorbance of the individual wells was measured at 450 nm using a Synergy H1 Microplate Reader. The concentrations of the secreted cytokines were calculated based on a standard curve. The inflammatory effects of MBJ were measured relative to the LPS-treated group.

### 2.10. Luciferase Reporter Assay

HEK 293 cells were seeded onto a 12-well plate at 70% cell confluency. The NF-κB or AP-1 promoter-containing luciferase reporter plasmids were purchased from Agilent Technologies (Santa Clara, CA, USA). The gWIZ-green fluorescent protein (GFP) plasmid was used as a control for normalization. The plasmids were transfected using polyethylenimine (Polysciences, Inc., Warrington, PA, USA) for 6 h at 37 °C. After 24 h, the transfected cells were treated with the indicated concentrations of MBJ (20, 50, 100, and 150 μg/mL) in the presence of phorbol 12-myristate 13-acetate. Following 24 h of incubation, the cells were lysed with Cell Culture Lysis Reagent (Promega Corporation, Madison, WI, USA), and their luciferase activities were measured using the Luciferase Assay System (cat no. E1500; Promega Corporation) according to the manufacturer’s instructions. Luminescence and fluorescence were measured using a Synergy H1 Hybrid Microplate Reader and analyzed using the Gen5 software version 1.11.5 (BioTek Instruments, Inc.).

### 2.11. Preparation of Total Cell Lysates

RAW 264.7 cells were pre-treated with MBJ (20, 50, 100, and 150 μg/mL) for 2 h and then stimulated with LPS (1 μg/mL) for 3 min (for IκBα, IRAK1, and TAK1), 15 min (for MAPKs), or 24 h (for iNOS and COX-2), and subsequently washed twice with cold PBS (pH 7.4). Cells were collected and lysed in lysis buffer containing 150 mM NaCl, 20 mM Tris-HCl (pH 8.0), 0.5% IGEPAL CA-630 (NP-40), 0.5% Triton X-100, 1 mM ethylenediaminetetraacetic acid, 1% glycerol, 2 mM phenylmethylsulfonyl fluoride (PMSF), 10 mM sodium fluoride (NaF), and 1 mM sodium orthovanadate (Na_3_VO_4_). The lysates were centrifuged at 15,814× *g* at 4 °C for 30 min. The supernatants were transferred to a new tube.

### 2.12. Immunoblotting Analysis

Immunoblotting analyses were performed as described in a previous study [31]. Briefly, protein concentrations were measured using a Bradford protein assay (Bio-Rad, Hercules, CA, USA) according to the manufacturer’s instructions. Aliquots of cell lysates were mixed with 5 × sodium dodecyl sulfate (SDS) sample buffer [12 mM Tris-HCl (pH 6.8), 0.4% SDS, 5% glycerol, 1% β-mercaptoethanol, and 0.02% bromophenol blue] and boiled at 100 °C for 5 min. The samples were then separated on 10% SDS-polyacrylamide gels and transferred to nitrocellulose membranes (GE Healthcare, Milwaukee, WI, USA). The membranes were blocked with 5% nonfat-dried skim milk in 1× Tris-buffered saline/0.05% Tween-20 (TBST) solution, followed by incubation at 4 °C overnight with primary antibodies. Each membrane was washed four times with 1× TBST and incubated with the appropriate secondary antibody at RT for 2 h. The target proteins were visualized using an enhanced chemiluminescence immunoblotting detection reagent (Pierce, Rockford, IL, USA). Protein levels were quantified by scanning the immunoblots and analyzing the images using the LabWorks software (UVP Inc., Upland, CA, USA). All protein levels were calculated relative to the LPS-treated group.

### 2.13. Animal Testing

Six-week-old male C57BL/6 mice were purchased from RaonBio (Gyeonggi-do, Korea) and maintained in a 12 h light/12 h dark cycle. All experiments were conducted according to internationally accepted practices for laboratory animal use and care, which are routinely implemented in US laboratories (NIH publication #85-23, revised in 1985). The experimental mice were orally administered either PBS or MBJ (100 mg/kg/day) once a day for 7 days. An hour after the final oral administration of the compound solution, the mice were intraperitoneally injected with LPS (10 μg/kg) and D-Gal (1 g/kg) to induce acute hepatitis inflammation. Control groups received the same volume of PBS intraperitoneally. Mice blood and spleen samples were collected 4 h after the induction of acute hepatitis, and total RNA was extracted from mouse liver tissues. Mice spleen tissues were homogenized using Accuzol total RNA extraction solution (Bioneer Corporation, Daejeon, Korea). Total RNA (1 μg) was reverse transcribed into cDNA as described above.

For the preparation of mouse tissue lysates, mice spleen tissues were lysed using lysis buffer [20 mM Tris-HCl (pH 8.0), 150 mM NaCl, 0.5% Triton X-100, 0.5% IGEPAL^®®^ CA-630, 1 mM EDTA, 1% glycerol, 1 mM PMSF, 10 mM NaF, and 1 mM Na_3_VO_4_]. Protein concentrations were determined via the Bradford protein assay (Bio-Rad Laboratories, Inc.) according to the manufacturer’s instructions. Aliquots of cell lysates were mixed with 5 × SDS sample buffer and boiled at 100 °C for 5 min.

### 2.14. Radical Scavenging (DPPH) Assay

DPPH radical scavenging activity assays were performed to evaluate the antioxidant activity of MBJ. An MBJ stock solution (200 mg/mL) was prepared by dissolving MBJ in DMSO. The stock solution was then diluted in 100% MeOH to concentrations of 20, 50, 100, 150, and 200 μg/mL, after which 100 μL of each MBJ concentration diluent was transferred to 96-well plates. Afterward, 100 μL of 0.2 mM DPPH solution was added and incubated for 30 min in a 37 °C incubator. After incubation, absorbance was measured at 517 nm using a Synergy H1 Hybrid Microplate Reader. The radical scavenging activity was then calculated as (Abs_sample_ − Abs_MeOH_)/Abs_MeOH_ and expressed as relative values. Quercetin (Sigma-Aldrich, St. Louis, MO, USA) and luteolin (Sigma-Aldrich, St. Louis, MO, USA) were used as positive controls.

### 2.15. Measurement of Serum Aspartate Transaminase (AST), Alanine Transaminase (ALT), and Blood Glutathione (GSH)/Glutathione Disulfide (GSSG) Ratio in an Acute Hepatitis Mouse Model

Serum samples were obtained by centrifuging the blood collected from six-week-old male C57BL/6 mice at 4000× *g* for 15 min. AST and ALT serum levels were detected by ELISA. GSH/GSSG ratios in the blood were detected using the EZ-Glutathione assay kit (DoGenBio, Seoul, Korea) according to the manufacturer’s instructions.

### 2.16. Statistical Analyses and Experimental Replicates

All experiments were performed at least three times. The results were reported as the means ± standard error of the mean (SEM). Pair-wise comparisons between treatments and the controls were conducted using Student’s t-test. Differences between experimental conditions were assessed using one-way ANOVA coupled with Dunnett’s multiple comparison test using Prism3.0 (GraphPad Software, San Diego, CA, USA). A *p*-value < 0.05 was considered statistically significant.

## 3. Results

### 3.1. Suppression of NO Production by MBJ in Macrophages

**Figure 1:**
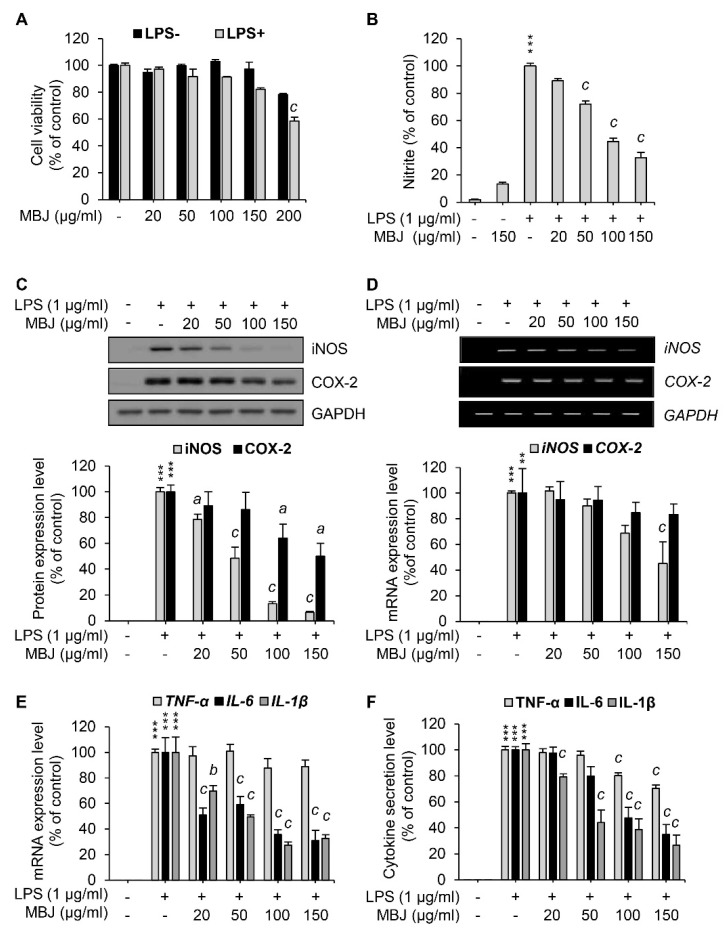
Inhibitory effects of MBJ on NO production, inflammatory gene expression, and cytokine secretion in RAW 264.7 macrophages. (**A**) RAW 264.7 macrophages were pre-treated with MBJ and then treated with or without LPS (1 μg/mL). The cell viability of each group was normalized to that of the MBJ-untreated group. The data represent the mean ± SEM; ^c^ *p* < 0.001 vs. the MBJ-untreated control group. (**B**) RAW 264.7 macrophages were pre-treated with MBJ and then stimulated with LPS (1 μg/mL) for 24 h. The NO secretion level of each group was measured relative to the LPS-only treated group. The amounts of secreted NO were calculated according to a standard curve developed from a standardized nitrite solution. The data represent the mean ± SEM; *** *p* < 0.001 vs. the non-treated group; ^c^ *p* < 0.001 vs. the LPS-treated and MBJ-untreated control group. RAW 264.7 macrophages were pre-treated with MBJ for 2 h and then stimulated with LPS for 24 h (**C**,**F**) or 3 h (**D**,**E**). (**C**) The protein levels of iNOS and COX-2 were analyzed by immunoblotting analysis. The protein levels were normalized to those of GAPDH. Relative expression levels of iNOS and COX-2 are represented as a bar graph (lower panel). (**D**) Levels of *iNOS* and *COX-2* mRNA were analyzed by RT-PCR. Relative mRNA levels of *iNOS* and *COX-2* are represented as a bar graph (lower panel). (**E**) *TNF-α, IL-6*, and *IL-1β* mRNA expression levels were analyzed by RT-qPCR. Relative expression levels of these mediators are represented as a bar graph. (**F**) The secreted levels of TNF-α, IL-6, and IL-1β in the culture media were measured using ELISA. The data represent the mean ± SEM; ** *p* < 0.01 and *** *p* < 0.001 vs. the non-treated group; ^a^ *p* < 0.05, ^b^ *p* < 0.01 and ^c^ *p* < 0.001 vs. the LPS-treated and MBJ-untreated control group.

The maximum non-cytotoxic concentration of MBJ in RAW 264.7 was evaluated using cell viability assays to minimize biases associated with cell death. RAW 264.7 cells were treated with various concentrations of MBJ with or without LPS stimulation. After treatment for 24 h, the cell viability of each group was measured. Cell viability assays using LPS-treated cells indicated that more than 80% of the cells survived up to 150 μg/mL MBJ (Figure 1A). Downstream assays are generally thought to be unaffected when the cell viability rates exceed 70% [32]. Therefore, RAW 264.7 cells were treated with no more than 150 μg/mL of MBJ in downstream experiments.

To investigate the anti-inflammatory effects of MBJ, the levels of NO (i.e., a crucial inflammatory mediator) were measured in LPS-treated cell culture media using the Griess reagent. NO production was inhibited by MBJ treatment in LPS-treated RAW 264.7 cells (Figure 1B). Specifically, NO production was significantly and dose-dependently suppressed when the cells were treated with MBJ. Therefore, our findings indicated that MBJ can suppress the inflammatory responses of LPS-treated macrophages.

### 3.2. Inhibitory Effects of MBJ on the Production of Inflammatory Mediators

To further analyze the anti-inflammatory effects of MBJ, the protein expression levels of COX-2 and iNOS were evaluated in RAW 264.7. When the cells were treated with MBJ for 2 h and then LPS for 24 h, the protein levels of iNOS were dose-dependently reduced, whereas COX-2 protein expression was partially inhibited by MBJ (Figure 1C). The relative expression of iNOS was almost entirely suppressed by 150 μg/mL of MBJ. On the other hand, the COX-2 expression level decreased to approximately 50% of the control group even at the maximum MBJ concentration. When the cells were treated with MBJ for 2 h and then LPS for 3 h, the transcriptional levels of *iNOS* were significantly decreased by MBJ, whereas those of *COX-2* decreased only slightly (Figure 1D), thus mirroring the patterns of their reduced protein levels (Figure 1C). Given that MBJ suppressed NO production, the mRNA levels of other inflammatory mediators were also measured to further evaluate the anti-inflammatory effects of MBJ. The transcriptional levels of *IL-1β* and *IL-6* were significantly suppressed by MBJ, whereas MBJ treatment only had a weak effect on *TNF-α* expression (Figure 1E). Similarly, the production of IL-1β and IL-6 was dramatically suppressed by MBJ, whereas that of TNF-α was only moderately inhibited (Figure 1F). These data suggest that the anti-inflammatory effects of MBJ are likely associated with the downregulation of inflammatory mediators.

### 3.3. Regulation of MAPK Phosphorylation and NF-κB Activation by MBJ

**Figure 2:**
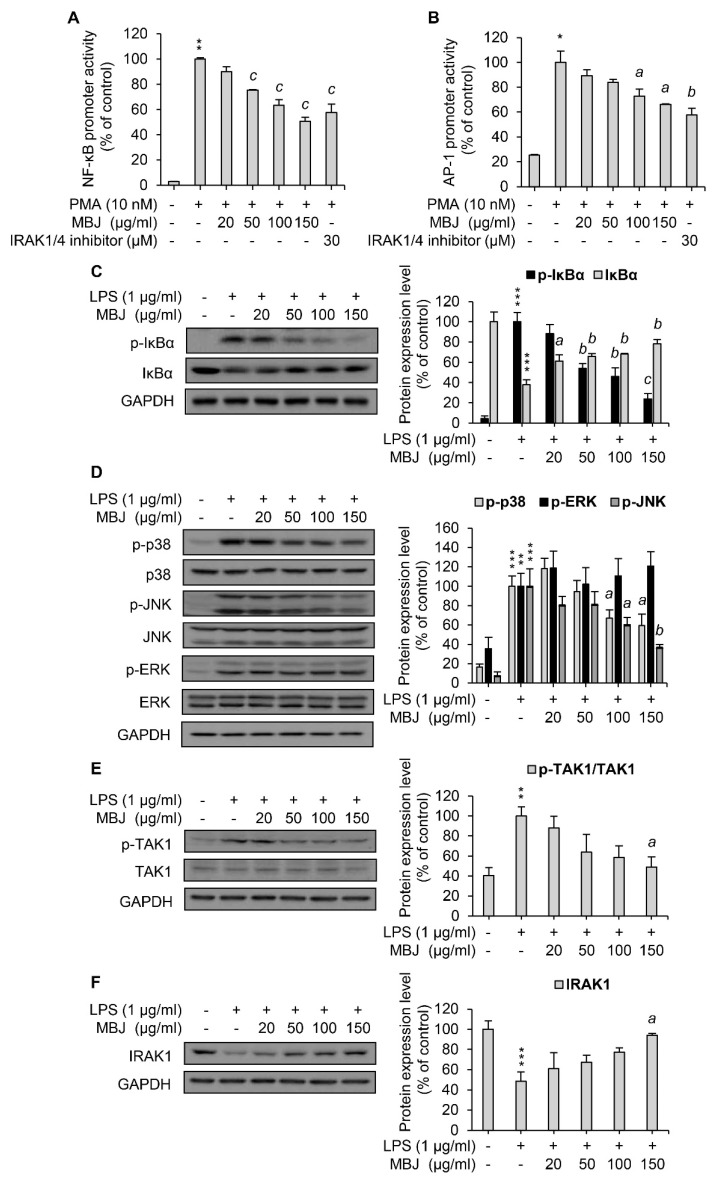
Suppression of the IRAK1-TAK1-NF-κB/MAPK pathways by MBJ. The promoter activity of (**A**) NF-κB and (**B**) AP-1 was assessed via luciferase assays using HEK 293 cell lysates. The data represent the mean ± SEM; * *p* < 0.05 and ** *p* < 0.01 vs. the PMA-untreated group; ^a^ *p* < 0.05, ^b^ *p* < 0.01 and ^c^ *p* < 0.001 vs. the PMA-treated and MBJ-untreated groups. (**C**–**F**) RAW 264.7 macrophages were pre-treated with MBJ for 2 h and stimulated with LPS for 3 min (**C**,**E**,**F**) or 15 min (**D**). The phosphorylated and total levels of (**C**) IκBα, (**D**) MAPKs, and (**E**) TAK1 were analyzed by immunoblotting analysis. Relative fold changes are presented as the ratio of the phosphorylated protein level to the total proteins after normalization to the LPS-treated and MBJ-untreated control group. (**F**) IRAK1 protein expression levels were analyzed by immunoblotting analysis. The data represent the fold changes relative to the non-treated control group. All protein levels were quantified using the LabWorks software and were normalized to the corresponding GAPDH levels. The data represent the mean ± SEM; ** *p* < 0.01, and *** *p* < 0.01 vs. the non-treated group; ^a^ *p* < 0.05, ^b^ *p* < 0.01 and ^c^ *p* < 0.001 vs. the LPS-treated and MBJ-untreated groups.

Given that MBJ inhibited the expression of inflammatory mediators in LPS-treated macrophages, we next sought to analyze the activities of inflammation-related signaling pathways. Reporter assays using the luciferase gene construct that contains NF-κB or AP-1- binding sites were employed. Because RAW 264.7 cells have poor transfection efficiency, HEK 293 cells were used for the transfection of reporter plasmids instead. In addition, PMA was used to induce the transcriptional activation of NF-κB and AP-1 for reporter assays in HEK 293 cells since the cells do not express TLR4. MBJ treatment inhibited NF-κB promoter activity to approximately 50% of the control in a dose-dependent manner (Figure 2A). AP-1 promoter activity, which is regulated by the MAPK pathway, was also repressed by MBJ (Figure 2B) but its repression was less pronounced than that of the NF-κB promoter. To further evaluate the activity of the NF-κB pathway, we first analyzed the phosphorylation level of IκBα, the inhibitor of NF-κB. When the cells were treated with MBJ, IκBα phosphorylation decreased in a dose-dependent manner (Figure 2C). Because phosphorylation of IκBα induces its degradation, a decrease in IκBα phosphorylation was observed when the level of total IκBα protein increased. Given that the inflammatory responses of LPS-treated RAW 264.7 cells are mediated by increased phosphorylation of p38, ERK, or JNK [33], the phosphorylation of each MAPK was also evaluated. The phosphorylation levels of p38 and JNK were decreased by MBJ treatment in a dose-dependent manner, whereas that of ERK was not suppressed (Figure 2D). Taken together, our findings suggest that the anti-inflammatory mechanisms of MBJ are driven by negative regulation of inflammation-inducing pathways, including the NF-κB, p38, and JNK pathways.

As the NF-κB, p38, and JNK pathways were downregulated by MBJ treatment, the phosphorylation of TAK1, a common upstream kinase of these pathways, was also evaluated. LPS-induced TAK1 phosphorylation was dose-dependently inhibited by MBJ in RAW 264.7 cells (Figure 2E). Moreover, IRAK1, a negative regulator of TAK1, was upregulated in the MBJ-treated group relative to the MBJ-untreated control group (Figure 2F). These data suggest that the suppression of inflammatory pathways by MBJ is mediated by the regulation of IRAK1-TAK1 in TLR4 signaling.

### 3.4. Effects of MBJ on Nrf2 Expression and Nuclear Translocation

**Figure 3:**
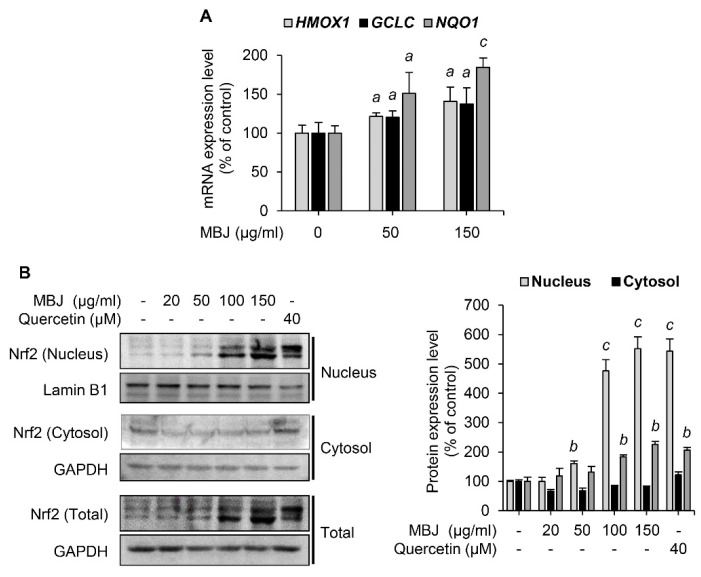
Effects of MBJ on the Nrf2 signaling pathways. (**A**) The relative mRNA expression levels of *HMOX1, GCLC*, and *NQO1* in RAW 264.7 macrophages were analyzed via RT-qPCR and presented as a bar graph. The data are shown as the mean ± SEM; ^a^ *p* < 0.05 and ^c^ *p* < 0.001 vs. the MBJ-untreated group. (**B**) Nrf2 protein levels in the nucleus were analyzed after subcellular fractionation of MBJ-treated RAW 264.7 macrophage cells. Quercetin was used as a positive control. All protein levels were quantified using the LabWorks software and were normalized to the corresponding Lamin B1 or GAPDH levels. The data represent the mean ± SEM; ^b^ *p* < 0.01 and ^c^ *p* < 0.001 vs. the non-treated groups.

Given that Nrf2 is an essential transcription factor that induces the expression of antioxidant enzymes [8], the effect of MBJ on Nrf2 was also investigated. Under oxidative stress, Nrf2 dissociates from Keap1, which sequesters Nrf2 in the cytoplasm and facilitates its degradation. The dissociated Nrf2 then translocates to the nucleus in response to the ROS signal [34]. The mRNA levels of Nrf2 target genes were measured to evaluate the antioxidant effects of MBJ. Our findings revealed that MBJ increased the mRNA expression levels of *HMOX1*, *GCLC*, and *NQO1* (Figure 3A). Nrf2 protein expression was increased both in the nucleus and total cell lysates (Figure 3B). These data suggest that the antioxidant effects of MBJ are mediated by the regulation of Nrf2 translocation and induction of its target genes.

### 3.5. Antioxidant and Anti-Inflammatory Effects of MBJ In Vivo

**Figure 4:**
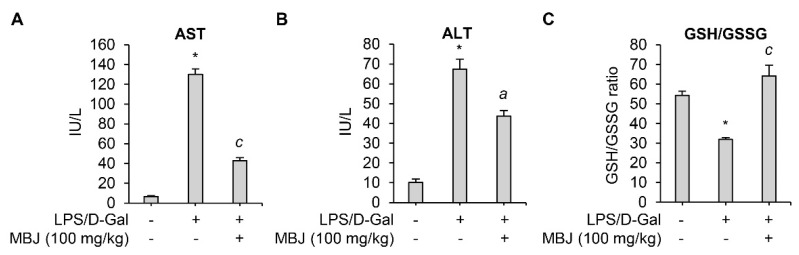
Antioxidant and anti-inflammatory effects of MBJ in mouse blood. (**A**) AST and (**B**) ALT levels in the mouse serum were analyzed. (**C**) The blood GSH/GSSG ratio was analyzed. The data are shown as the mean ± SEM; * *p* < 0.05 vs. the LPS/D-Gal-untreated group; ^a^ *p* < 0.05 and ^c^ *p* < 0.001 vs. the LPS/D-Gal-treated and MBJ-untreated group. IU/L, International units per liter.

When co-exposed to LPS and D-Gal, animals develop lethal liver injury mimicking hepatitis [35]. The inflammatory cytokines and ROS produced by LPS in the injured liver led to high levels of liver enzymes such as AST and ALT in serum [36,37,38,39]. Therefore, serum levels of AST and ALT in an LPS/D-Gal-induced acute hepatitis mouse model were measured to evaluate the anti-inflammatory effect of MBJ in vivo. LPS/D-Gal-induced AST and ALT levels were significantly suppressed by oral administration of MBJ (100 mg/kg) (Figure 4A,B). In addition, previous studies have linked LPS exposure with decreased GSH levels due to oxidative stress [40,41]. Depletion of GSH is related to an increase in GSSG concentration and a lower GSH/GSSG redox ratio under oxidative stress conditions [39]. Therefore, GSH/GSSG ratios were analyzed to evaluate the antioxidant properties of MBJ. The GSH/GSSG ratio was significantly decreased in the blood of LPS/D-Gal treated mice but recovered after MBJ administration (100 mg/kg) (Figure 4C). These results suggest that MBJ plays a role in suppressing both inflammation and oxidative stress in vivo.

**Figure 5:**
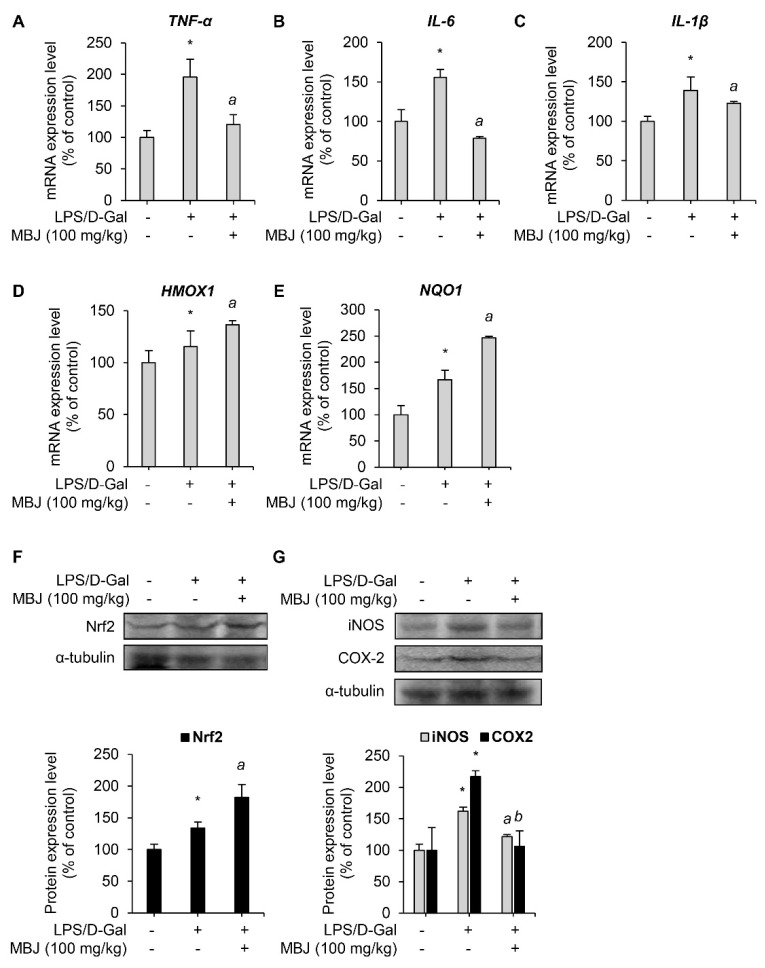
Antioxidant and anti-inflammatory effects of MBJ in vivo. (**A**–**E**) The mRNA expression levels of target genes in the spleen of mice were analyzed via RT-qPCR. The relative mRNA expression levels of inflammatory mediators (*TNF-α*, *IL-6*, and *IL-1β*) or antioxidant mediators (*HMOX1* and *NQO1*) are presented as a bar graph. The data are shown as the mean ± SEM; * *p* < 0.05 vs. the LPS/D-Gal-untreated group; ^a^ *p* < 0.05 vs. the LPS/D-Gal-treated and MBJ-untreated group. Mice spleen expression levels of (**F**) the Nrf2 protein and (**G**) the iNOS and COX-2 proteins were analyzed by immunoblotting. All protein levels were quantified using the LabWorks software and were normalized to the corresponding α-tubulin levels. The data represent the mean ± SEM; * *p* < 0.05 vs. the LPS/D-Gal-untreated group; ^a^ *p* < 0.05 and ^b^ *p* < 0.01 vs. the LPS/D-Gal-treated and MBJ-untreated groups.

The mRNA levels of inflammatory mediators and antioxidant regulators in the spleen of the experimental mice were measured to evaluate the anti-inflammatory and antioxidant effects of MBJ in vivo. The mRNA expressions of the inflammatory mediators, *TNF-α*, *IL-6*, and *IL-1β*, were suppressed by MBJ (100 mg/kg) (Figure 5A–C). Furthermore, the mRNA expression levels of the Nrf2 target genes, *HMOX1* and *NQO1,* increased upon MBJ administration (Figure 5D,E). Moreover, MBJ treatment decreased iNOS and COX-2 protein levels but increased Nrf2 protein levels in mouse spleens (Figure 5F,G). These data suggest that the suppression of the inflammatory and antioxidant responses in vivo by MBJ are also mediated by the regulation of inflammatory mediators and Nrf2.

### 3.6. HPLC and DPPH Analyses of MBJ

**Figure 6:**
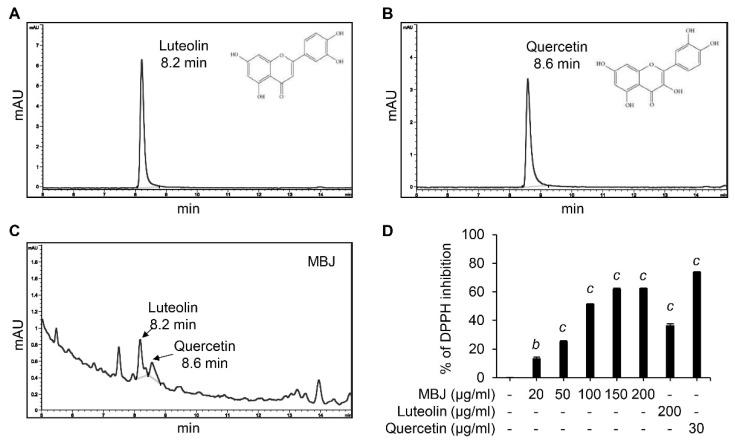
HPLC and DPPH assays. HPLC analysis data of (**A**) luteolin, (**B**) quercetin, and (**C**) MBJ. The chemical structures of luteolin and quercetin are illustrated in the top right corner. (**D**) The antioxidant effect of MBJ was analyzed using DPPH assays. Luteolin (200 μg/mL) and quercetin (30 μg/mL) were used as positive controls. The data are shown as means ± SEM. ^b^ *p* < 0.01 and ^c^ *p* < 0.001 vs. the non-treated control group.

Our data indicated that MBJ exhibits antioxidant and anti-inflammatory properties. Interestingly, quercetin and luteolin are known to have these effects [42,43] and are the major phytoconstituents of *B. javanica* [44,45,46,47,48]. Therefore, HPLC and DPPH analyses were conducted to verify the presence of these compounds in MBJ. HPLC analyses indicated that the retention time of luteolin and quercetin peaked at 8.2 min and 8.6 min, respectively (Figure 6A,B). When MBJ was analyzed under the same conditions, the major peaks of luteolin and quercetin were observed at the same retention times (Figure 6C), suggesting that MBJ contains these active compounds. In addition, MBJ exhibited dose-dependent antioxidant properties when DPPH assays were conducted with MBJ and the aforementioned components (Figure 6D), suggesting that the antioxidant effects of MBJ were mainly due to its luteolin and quercetin contents.

## 4. Discussion

Uncontrolled or abnormal inflammatory responses have been linked to several diseases, including asthma, rheumatoid arthritis, autoimmune diseases, and even cancer [49,50,51,52]. These inflammatory responses are typically ameliorated by chemical-based medications, which often cause side effects [53,54,55]. Natural plant extracts have been found to possess antioxidant activity and inhibit the production of inflammatory mediators [56]. Various types of *B. javanica* extracts were reported to contain tannins, β-amyrin, betulinic acid, friedelan-3α-ol, epifriedelinol, friedelin, luteolin, quercetin, beta-sitosterol, stigmasterol, and ursolic acid [44,45,46,47,48]. Particularly, some of these phytochemicals were reported to have antioxidant or anti-inflammatory properties [57,58,59,60]. There is a growing need to understand the mode of action of these phytochemicals to avoid unwanted side effects, and therefore the molecular mechanisms through which MBJ exerts its antioxidant and anti-inflammatory properties were investigated in this study.

Increased NO levels have been associated with inflammatory diseases, and most anti-inflammatory drugs suppress NO production in macrophages [61]. These suppressed NO levels coincided with the inhibition of iNOS at the transcriptional level upon MBJ treatment. Interestingly, the transcription level of COX-2 did not decrease as much as that of iNOS. Because COX-2 expression is mediated by several pathways, including the ERK, p38, NF-κB, and protein kinase C pathways [62], the differential regulation of iNOS and COX-2 expression by MBJ may be the result of specific signaling pathway inhibitions. Although MBJ inhibited the NF-κB and MAPK pathways, NF-κB activity was more suppressed than that of AP-1. This differential inhibition of pro-inflammatory mediators might provide insights into the mechanisms by which anti-inflammatory reagents regulate intracellular pathways.

Inflammatory cytokines not only mediate inflammatory responses but also enhance the production of NO by inducing the expression of iNOS, thereby promoting chronic inflammatory diseases such as rheumatoid arthritis and asthma [21,63]. Cytokine secretion levels were decreased upon MBJ treatment, which coincided with altered mRNA expression levels. Furthermore, the expression levels of inflammatory cytokines are directly regulated by the activities of NF-κB and AP-1, which bind to the gene promoter region [64]. Interestingly, the inhibitory effect of MBJ on the AP-1 promoter activity was less pronounced than its effect on the activity of the NF-κB promoter. MAPK phosphorylation analyses indicated that the phosphorylation of both p38 and JNK were decreased by MBJ, whereas the level of phosphorylated ERK remained largely unchanged. These results indicate that MBJ targets only p38 and JNK, but not the ERK pathway. Given that AP-1 activity is regulated by ERK as well as p38 and JNK [65,66], a decrease in the suppression of AP-1 activity might result in differences among IL-6 and TNF-α secretion levels. Our previous study indicated that TNF-α production was effectively inhibited when the NF-κB, JNK, p38, and ERK pathways were all suppressed [31]. Other studies have demonstrated that betulinic acid inhibited the p38 and JNK pathways [67]. Betulinic acid is one of chemical constituents of MBJ and therefore may be responsible for the selective inhibition of the MAPK pathways by MBJ. In other words, the selective inhibition of the MAPK pathways by MBJ may be attributed to the chemical composition of MBJ. TAK1, a common upstream kinase of the NF-κB, p38, and JNK pathways [68], was suppressed by MBJ, which coincided with a decrease in the phosphorylation of p38, JNK, and IκB. The phosphorylation of TAK1 is induced when IRAK1 of the TLR signaling complex is rapidly phosphorylated, ubiquitinated, and then degraded [11,12]. Therefore, β-TrCP-induced IRAK1 degradation is a critical step for TAK1-dependent NF-κB activation [69]. MBJ treatment increased IRAK1 protein levels in a dose-dependent manner, indicating that MBJ blocked IRAK1 degradation. Taken together, our findings illustrate the sequential effects of MBJ via the suppression of IRAK1 degradation, which eventually downregulates the expression of inflammatory mediators both in vitro and in vivo.

The mechanism through which MBJ exerts its antioxidant properties was dependent on the translocation of Nrf2 to the nucleus. Nrf2 is a pivotal regulator of detoxication enzymes and induces antioxidant responses to eliminate ROS [8]. MBJ stabilized Nrf2 and promoted Nrf2 translocation to the nucleus, which protects Nrf2 from ubiquitination-dependent degradation via sequestration in the cytoplasm. Impaired Nrf2 translocation causes severe diseases such as Friedreich Ataxia, which causes neuronal death and muscle movement problems [70]. Nrf2 agonists show neuroprotective effects in damaged HT22 cells [71]. Based on previous reports and the present study, MBJ may be a highly effective antioxidant, as it induces Nrf2 translocation and the expression of antioxidant genes that protect cells from oxidative stress.

## 5. Conclusions

**Figure 7:**
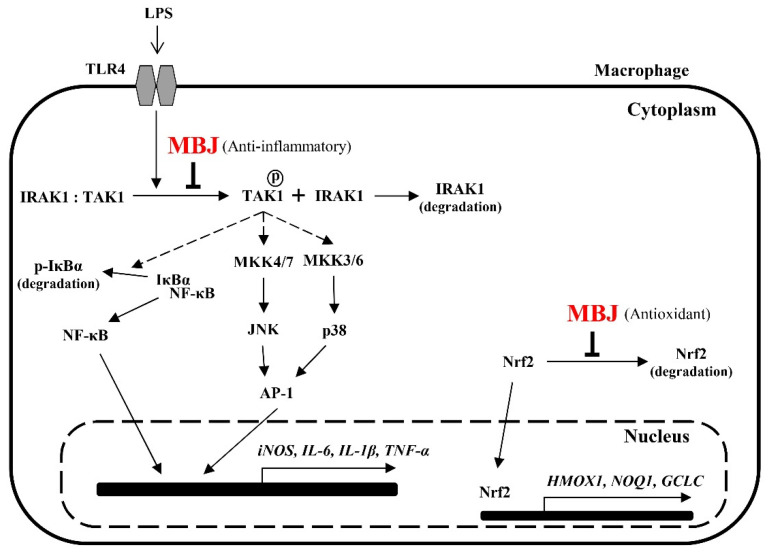
Molecular pathways that mediate the antioxidant and anti-inflammatory effects of MBJ in RAW 264.7 cells. MBJ may affect inflammatory mediators by regulating signal transduction from TLR4 to IRAK1 and inhibiting the activation of the NK-κB and MAPK signaling pathways. Further, MBJ may affect oxidative stress regulation by inducing the translocation of Nrf2 to the nucleus, thereby inducing downstream gene expression.

MBJ negatively regulates the production of pro-inflammatory mediators and triggers Nrf2 translocation. Furthermore, the NF-κB, p38, and JNK signaling pathways are inhibited by MBJ. The anti-inflammatory mechanisms of MBJ are mediated by the regulation of TLR signaling complexes, including IRAK1 and TAK1 (Figure 7). Given that the inhibition of TLR signaling from the uppermost cascade has the beneficial effects of suppressing the inflammatory responses, our results suggest that MBJ could be applied to improve inflammatory and redox imbalances in different types of pathologies.

## Data Availability

Data is contained within the article.
